# Antioxidants, Dietary Fatty Acids, and Sperm: A Virtual Reality Applied Game for Scientific Dissemination

**DOI:** 10.1155/2019/2065639

**Published:** 2019-11-11

**Authors:** Giulia Collodel, Maurizio Masini, Cinzia Signorini, Elena Moretti, Cesare Castellini, Daria Noto, Simona Mattioli, Vincenzo Santalucia, Stefano Baraldi, Luca Maurizio Lusuardi, Matteo Sirizzotti, Alessandro Innocenti

**Affiliations:** ^1^Department of Molecular and Developmental Medicine, University of Siena, Policlinico Le Scotte, Viale Bracci, 14, 53100 Siena, Italy; ^2^Department of Social, Political and Cognitive Sciences, University of Siena, Palazzo San Niccolò, via Roma, 56, 53100 Siena, Italy; ^3^Department of Agricultural, Food and Environmental Sciences, University of Perugia, Borgo XX, Giugno, 74, 06121 Perugia, Italy

## Abstract

Fatty acid (FA) profile appears to be critical to infertility, and the effects of dietary FAs on sperm FA content are a current focus of studies in the field of nutrition and reproduction. Starting from a validated “OXISTRESS” model in which modification of FA content results to influence reactive oxygen species, antioxidants, isoprostanes, cytokines, sperm kinetic, and acrosome reaction, we developed a virtual reality game where the player, in order to improve the health of some virtual spermatozoa, is called to take dietary choices and then discover their consequences on the main biological aspects. In the LabVR of the University of Siena, a team of VR environment designer and developer used Unity development engine to make the experience run on Oculus Quest and a wireless 6DOF (six degrees of freedom of movement in 3D space) VR Headset. In the game, the player is immersed in the epididymis and observes closer how dietary n-3 may change the sperm plasma membrane and consequently modify sperm traits. A simulation game in the virtual reality may represent a tool to give greater visibility to scientific data in the relevance of appropriate dietary habits in the human health.

## 1. Introduction

Understanding the molecular aspects of spermatogenesis would provide very interesting information on reproductive biology.

The phospholipid fraction of mammalian sperm membrane consists of about 50% of long chain-polyunsaturated fatty acids (LC-PUFAs), which influences many aspects of sperm physiology. LC-PUFAs play a key role in the sperm membrane fluidity and susceptibility to lipid peroxidation, sperm maturation, motility, and acrosome reaction [1].

Fatty acid (FA) profile appears to be critical to infertility, and the effects of dietary FAs on sperm FA content are a current focus of studies in the field of nutrition and reproduction [2]. Dietary FAs influence sperm FA profiles, and it seems that sperm FA contents are most sensitive to dietary n-3 PUFAs [3]. The potential application of a dietary strategy in relation to the improvement of FA profile of sperm and fertility has been investigated.

We are testing, using rabbit buck as an animal model, the effect of dietary plans with different n-6/n-3 PUFA ratio on semen quality.

It is widely recognized that several aspects affect the oxidative stress of semen such as infection, varicocele, environmental conditions, genetics, chronic inflammation, and diet.

**Figure 1:**
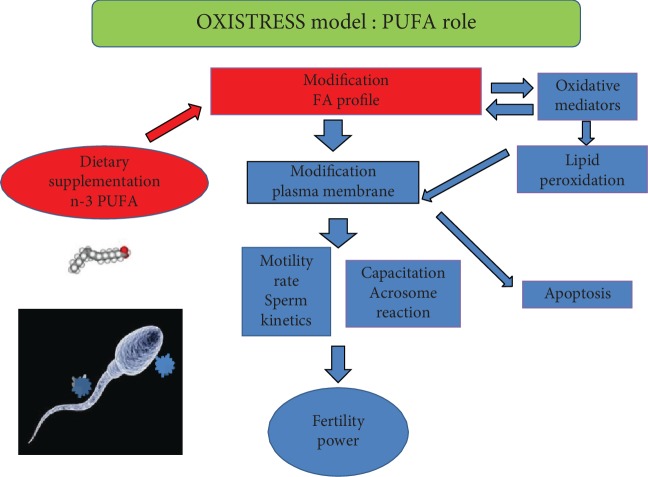
OXISTRESS model. Modification in fatty acid (FA) profile positively or negatively influences sperm function; n-3 supplementation positive influences sperm parameters.

Starting from experimental results in terms of FA profile, we developed and validated an OXISTRESS model (see Figure 1) in which modification of FA content results to influence reactive oxygen species, different index molecules (antioxidants, isoprostanes, and cytokines), sperm kinetic, and acrosome reaction.

Since it is difficult to imagine the composite effect of a dietary FA intervention in the sperm cell and to observe the effect on its function and to attract a broader community, we tried to find an alternative and effective way to reach a not specialized audience in order to maximize the benefit of the research with minimum delay.

Along with production of papers addressed towards the scientific community, we decided to design and develop, in collaboration with the laboratory of virtual reality (LabVR) of the University of Siena, a VR application which uses a plain language and appropriate information level to target an audience, which is not preengaged in the subject matter nor waiting for the output of the research.

The VR application is under development in the LabVR by a team of VR environment designer and developer, which use Unity development engine to make the experience run on Oculus Quest, a wireless 6DOF (Six degrees of freedom of movement in 3D space) VR Headset, released recently.

Digital applications and digital game environment have become important tools for education and training, and increasingly evidence-based theories are found about the educational benefits of computer games related to improvement of learning outcome thanks to value-adding features [4], improvement of general cognitive skills [4], and enhancement of motivation towards the learning subject [5, 6].

When games are compared to conventional media, there are substantially no results pointing that games are generally inferior to traditional instruction [4]; indeed, especially when we look at successful case studies in health [7] and food education [8], we can see “some reason to suspect that games can be as effective or more effective than traditional instruction for certain instructional domains and objectives” [4].

Virtual reality (VR) offers several advantages including a more realistic, lifelike environment that may allow subjects to “forget” they are being assessed, allowing a better participation and an increased generalization of learning [9]. It provides a simulated artificial environment in which one's actions determine sensory stimulation and is a valuable tool for investigating a wide spectrum of behaviors from sensorimotor interactions to spatial navigation and cognition [10].

In neuroscience, the VR system has been used to evaluate the patient's multimodal integration and to aid rehabilitation of cognitive abilities [11]. Specifically, in stroke rehabilitation, VR has been considered an important patient-centered tool because of its characteristics, such as contextualized environments and task-oriented training [12]. VR has been also used to treat various psychiatric disorders [13]. Recently, authors report that public speaking anxiety decreases within repeated VR training sessions [14] and VR offers promising results in sex therapy, particularly for the treatment of genital pain disorders in which anxiety plays a significant etiological role [15]. Regarding biology, a life sciences software company developed a VR visualization tool, called InViewR and ChimeraX; another molecular visualization tool for proteins was projected at the University of California [16].

These experiments show some different possibilities of VR application in repro field; however, we decided to move beyond the immersive visualization of microorganisms and to give priority to the interaction of the user with the dynamics of living systems.

## 2. OXISTRESS VR Applied Game

We aimed to build a compelling experience in which the players would discover the behaviors of the repro system subject of the research through the immersion and interaction within that system. We also wanted them to learn, during play, the effects of food-related habits on reproductive health. In collaboration with the colleagues of the LabVR, we found a main problem to deal with: for the game to be effective as a learning tool, we would have needed to design a conflict (a problem to solve) which would have requested to the player the development of that precise skill that we wanted her to learn. For example, a “sperm race” would have been a useless activity since it would train the player on skills like spatial attention and hand-eye coordination which are not pertinent with our learning objectives. Accordingly, we imagined a game where the player, in order to improve the health of some virtual spermatozoa, is called to take dietary choices and then discover their consequences on the main biological aspects. In the game, the player is immersed in the epididymis and observes closer how dietary n-3 may change the sperm plasma membrane and consequently modify sperm traits.

Through the dietary choices, the user can modulate the quantity and the interaction of FAs, antioxidants, and ROS and is nudged to understand their role in the system in order to take informed choices which would be resonant and, at the end, easy to remember.

By this design, the relevance of a correct diet would have directly visualized and demonstrated in male reproduction but it leaves space for another issue: we know the dietary choices are just one of the many factors that determinate the health state of cell membranes. We also know that a game design rule is for the player to be engaged; the player must perceive the critical actions to progress in the game. Would the player feel a sense of agency if the choices would not have been decisive to reach the game goal?

To overcome this obstacle, we came to conceive a fictional scenario where player's choices have immediate and strongly exaggerated consequences and any other variable, even those being decisive in the real world, becomes irrelevant.

The sci-fi nature of the scenario (the control of sperm quality in a group of astronauts has to colonize a new planet, which is protected by a hyperconsequential force field) would have helped us to justify at a game level the focus on the sperm quality, which would be hard to be turned into an “epic goal” with a different narrative setting.

Here, we came to the last doubt we had to solve: are we going too far? Will this game be close enough to reality to be still useful for his dissemination scope?

To answer the question, we find and illuminate a quote from the pioneer environmental scientist Donella Meadow [17]: “Model utility depends not on whether its driving scenarios are realistic, but on whether it responds with a realistic pattern of behavior”.

Concerning the player point of view, we are pretty convinced that as long as we provide a plausible conflict to deal with and the space for meaningful choices (for the concept of meaningful play see Salen and Zimmerman [18]), we can rely on the players' suspension of disbelief for expecting them to engage in the activity by taking its outcomes very seriously, as they usually do in any game.

In conclusion, this new approach represents a facilitation in dissemination of the paramount influence of nutrients in the sperm quality and consequently in male reproduction. By the same game, the role of nutrients could be emphasized in different physiological and pathological conditions and could also represent a model to implement with other effects (environmental, stress, pathological situation, smoke etc.).

A simulation game in the virtual reality may represent a tool to give greater visibility to scientific data in the relevance of appropriate dietary habits in the human health.

## Data Availability

The data used to support the findings of this study are included within the supplementary information file.

## Supplementary Materials

Supplementary Materials In the “Supplementary material,” a detailed description of the game, to which the manuscript refers, is provided. The principles of the game design are reported and a map of the game is detailed, step by step. The described game is to be intended as a game-based learning.

## References

[1] Esmaeili V., Shahverdi A. H., Moghadasian M. H., Alizadeh A. R. (2015). Dietary fatty acids affect semen quality: a review. *Andrology*.

[2] Wathes D. C., Abayasekara D. R. E., Aitken R. J. (2007). Polyunsaturated fatty acids in male and female reproduction. *Biology of Reproduction*.

[3] Nassan F. L., Chavarro J. E., Tanrikut C. (2018). Diet and men's fertility: does diet affect sperm quality?. *Fertility and Sterility*.

[4] Mayer R. E. (2019). Computer games in education. *Annual Review of Psychology*.

[5] Amory A., Naicker K., Vincent J., Adams C. (1999). The use of computer games as an educational tool: identification of appropriate game types and game elements. *British Journal of Educational Technology*.

[6] Roussou M. (2004). Learning by doing and learning through play. *Computers in Entertainment*.

[7] Ferguson B. (2012). The emergence of games for health. *Games for Health Journal*.

[8] Petros P., Georgios F. Educational applications of serious games: the case of the game food force in primary education students.

[9] Bioulac S., de Sevin E., Sagaspe P. (2018). What do virtual reality tools bring to child and adolescent psychiatry?. *L'Encephale*.

[10] Thurley K., Ayaz A. (2017). Virtual reality systems for rodents. *Current Zoology*.

[11] Bohil C. J., Alicea B., Biocca F. A. (2011). Virtual reality in neuroscience research and therapy. *Nature Reviews. Neuroscience*.

[12] Palma G. C. D. S., Freitas T. B., Bonuzzi G. M. G. (2017). Effects of virtual reality for stroke individuals based on the International Classification of Functioning and Health: a systematic review. *Topics in Stroke Rehabilitation*.

[13] Oing T., Prescott J. (2018). Implementations of virtual reality for anxiety-related disorders: systematic review. *JMIR Serious Games*.

[14] Takac M., Collett J., Blom K. J., Conduit R., Rehm I., de Foe A. (2019). Public speaking anxiety decreases within repeated virtual reality training sessions. *PLoS One*.

[15] Lafortune D., Dion L., Renaud P. (2019). Virtual reality and sex therapy: future directions for clinical research. *Journal of Sex & Marital Therapy*.

[16] Matthews D. (2018). Virtual-reality applications give science a new dimension.. *Nature*.

[17] Meadows D. (2009). *Thinking in Systems: A Primer*.

[18] Salen K., Zimmerman E. (2003). *Rules of Play: Game Design Fundamentals*.

