# Supplementary material for: Antioxidants, Dietary Fatty Acids, and Sperm: A Virtual Reality Applied Game for Scientific Dissemination
Source: Oxid Med Cell Longev. 2019 Nov 11;2019:2065639. doi: 10.1155/2019/2065639 (PMC6877968; doi:10.1155/2019/2065639)
Supplement: Supplementary Materials — In the “Supplementary material,” a detailed description of the game, to which the manuscript refers, is provided. The principles of the game design are reported and a map of the game is detailed, step by step. The described game is to be intended as a game-based learning. [file 2065639.f1.docx]

The data used to support the findings of this study are included within the supplementary information file(s).

The authors give information about the project presented in the paper, other data used to support the findings of this study have not been made available because the final application is included in a platform.

**The game**

OxiStress is a narrative-driven educational game.

As we said, the game is built around a story that allows us to isolate the biological system we want to talk about (diet and semen quality) and, also, to stage a fictitious context in which the individual punctual choices (what to eat) have powerful outcomes  in the simulation, as if they were consolidated habits.

**Narrative setting**

The player accidentally finds himself in radio contact with the captain of a spaceship in an interplanetary colonization mission, during a critical moment: the crew has found a planet hospitable to life, but the planet is protected by a hyper-bio-consequential field, i.e. the biological consequences of every action taken while passing in the atmosphere are amplified to exaggeration.

The captain asks the player for instructions on how to behave during the transit in the atmosphere to make a diet that maximizes the quality of the astronauts' semen (then the fertility).

The player finds out that every single choice has huge biological consequences, that they can observe closely thanks to the presence of a VR probe in the testes of an astronaut "reference subject".

The game ends with the landing of the spaceship on the planet. According to the choices made during the descent, the colonization mission will have more or less success.


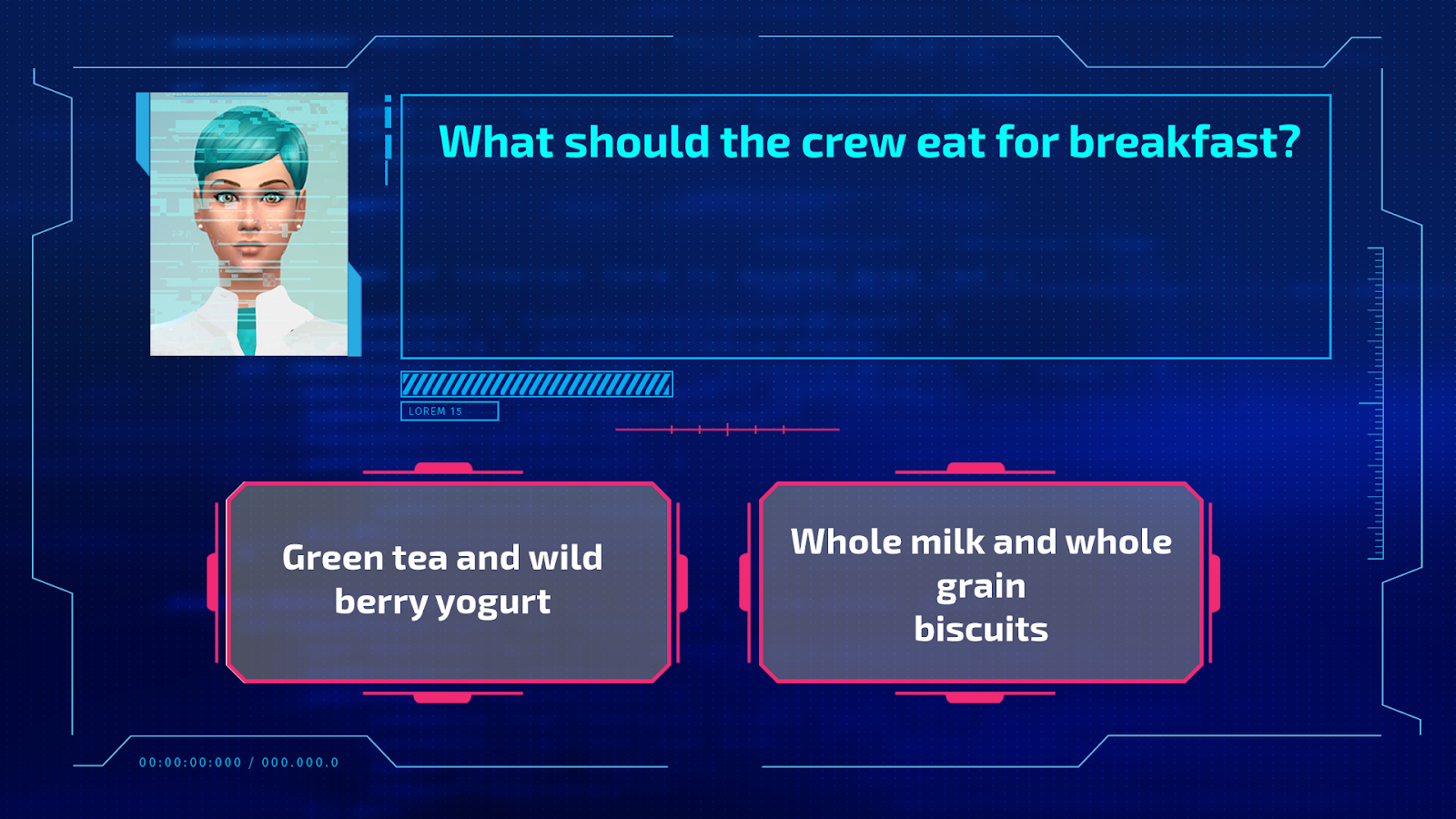
Dialogue options mockup

**GamePlay**

The descent to the planet lasts one day. The opinion of the player is required for several activities of the crew of that day, particularly on what to eat for breakfast, lunch, and dinner.

The player expresses his choices by selecting one of several multiple answers. According to his choices, the player sees that the fertility score of the reference subject changes, following an improvement or a deterioration of the semen quality.

The player is not immediately informed about why the fertility score increases or decreases.

Indeed, she can lead virtually the probe in the testes (3d reconstruction of the seminiferous tubules during spermatogenesis) and point the laser at the active areas to get information on the situation (ex. "reduced sperm motility" "detected high presence of reactive oxygen species (ROS)") to find out the relationship between choices and the change of the fertility score.


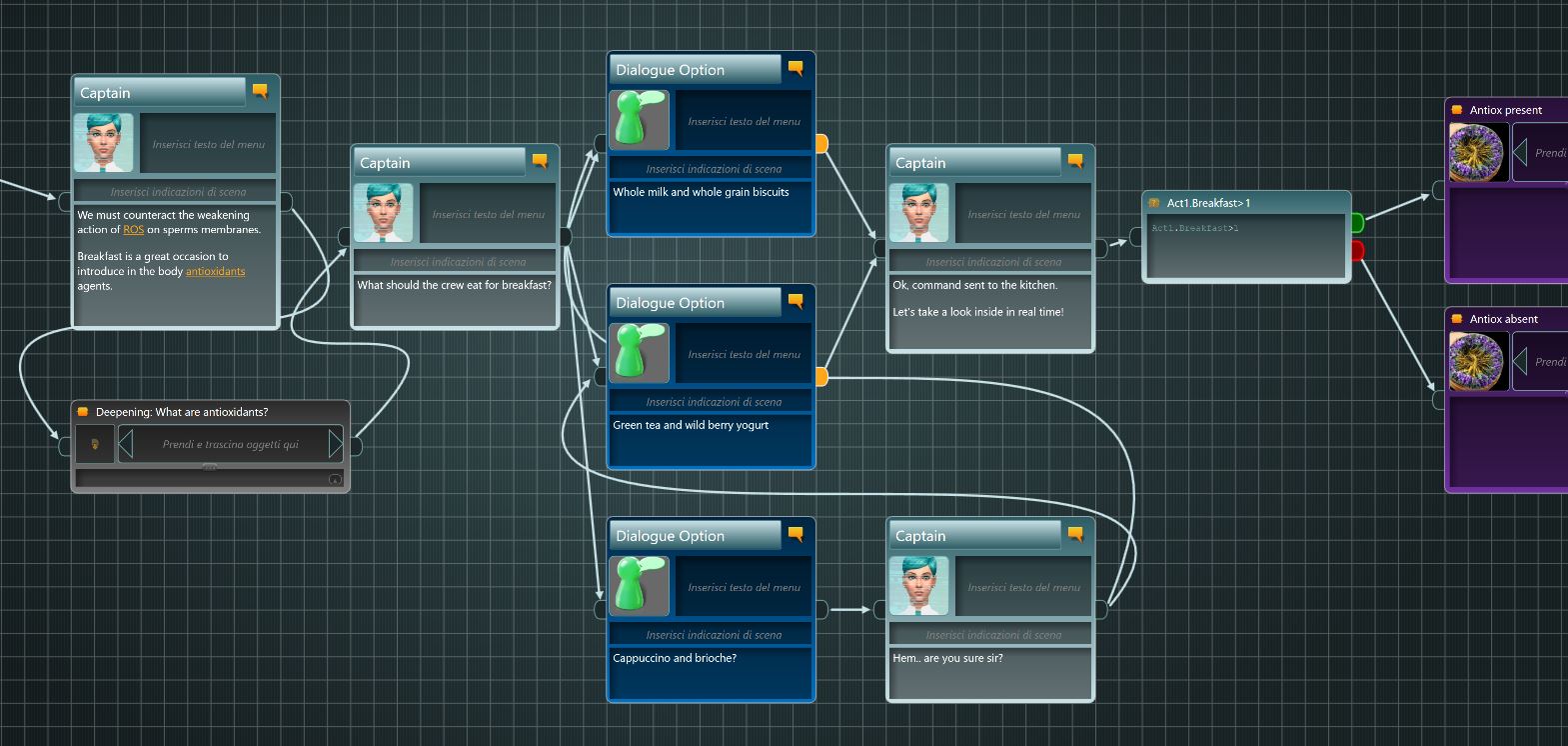


A dialogue options example

The player, controlling the probe, can also collect antioxidant molecules or omega 3 and omega 6 fatty acids (FA), which abound or run low according to the dietary choices made.

If she made the correct food choices, the player can:

- Shoot antioxidants on ROS molecules to counteract them,
- Throw FA molecules on sperm to improve their membrane composition.

Besides having an intuitive representation of the oxidant/antioxidants/FA system, the player will find out that there are different types of FA (reference to DHA and EPA) and that each type is more or less abundant and tends to fit into the sperm membrane at the level of the tail or in the post acrosomal region.

The player's progress is expressed by the fertility score which is the average of 3 basic sperm parameters that can be read by the player in the VR scenes:

1. Progressive motility (movement of the 3D sperm)
2. Morphology (regularity of the shape of the 3D sperm)
3. Vitality (smoothness and shininess of the 3D sperm shader)

Each parameter can have a negative, intermediate or good value.


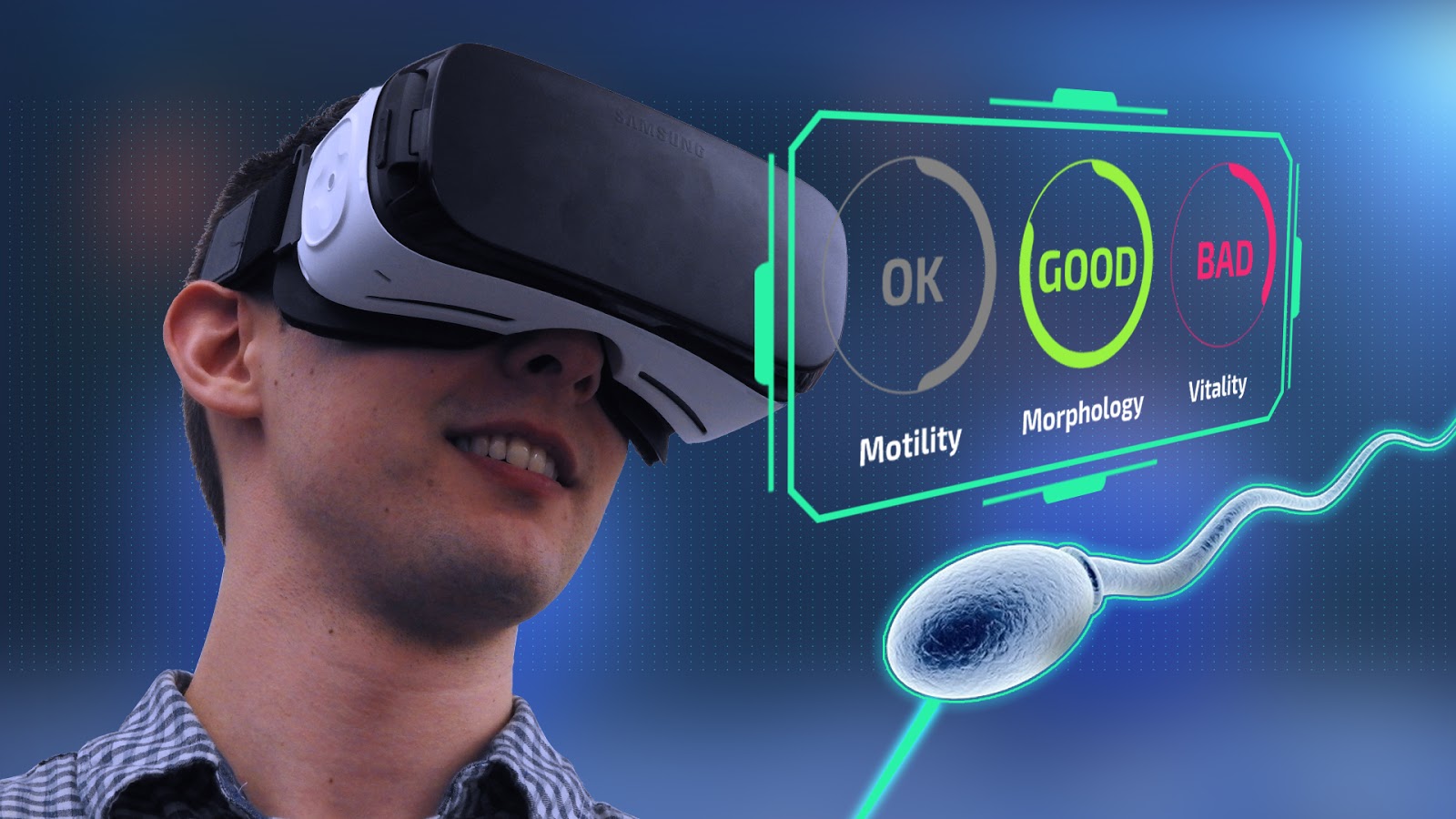


Concept image

**Main events**

| Event | Information | Meta information | Fun factor |
| --- | --- | --- | --- |
| ATTO 1 - SETUP | | | |
| Introductive dialogue | A correct diet can improve male fertility (better semen quality)  Spermatogenesis is a cyclical process that lasts lifetime. | The game is about diet and fertility  In this game, I can make choices via dialogue options. | *"What's happening?"* |
| Tutorial VR (1) | Sperm are formed in the seminiferous tubules in the testes.  Fertility is related to sperm progressive motility, morphology and vitality. | I will explore in VR the inside of a seminiferous tubule. | *“Cool, I’m driving a micro spacecraft”* |
| Tutorial VR (2) | In semen there are ROS; if their quantity is too high, sperm get damaged. | Ponting the laser I can get information about what I see.  ROS make sperm motility, morphology and vitality worse.  I’m going to fight ROS | *“I want to terminate these bad guys!”* |
| Breakfast dialogue | ROS can be counteracted by antioxidants.  Food can contain antioxidants. | In the game, I will choose between several dietary options. | *“How much I already know about this topic?”* |
| Breakfast VR | The foods I choose are poor/rich of antioxidants. | If I pick the right dishes, I’ll find antioxidant in semen.  I can collect and shoot antioxidant on ROS to counteract them. | *“Yay, shooting!”* |
| ACT 2 - CONFRONTATION | | | |
| Snack dialogue |  | There’s always at least one right option. The others will be partially or totally wrong | *“Ok, I got it”* |
| Snack VR | The foods I choose are poor/rich of antioxidants. |  | *“I’m learning!”* |
| Activity dialogue | Besides diet, there are other factors that can change the ROS/antioxidant balance. |  | *“It’s getting easy for me...”* |
| Activity VR | Intense physical activity increases ROS production.  UV radiation increases ROS production.  ROS can induce programmed death cell (apoptosis). | Making the right choice is no small matter. | *“Ops, I’m in trouble...”* |
| Launch dialogue (1) |  |  | *“I can do this”* |
| Launch VR (1) | Antioxidants neutralize ROS but they haven’t direct positive effects on sperm cell membranes. | ROS and antioxidants aren’t the only important agents of the system. | *“I’m approaching the central point...”* |
| Launch dialogue (2) | Introducing antioxidants isn't a enough improvement to diet in order to maximize the semen quality. |  | *“Here I am, in the central point”* |
| Launch VR (2) | Some foods contain omega 3 and omega 6 FA.  FA may modify the lipid membrane composition improving the fluidity and influencing sperm motility, acrosome reaction and capacitation. | FA improve sperm motility and vitality. | *“Hey, I’m learning things here!”* |
| ACT 3 - RESOLUTION | | | |
| Unexpected event dialogue |  |  | *“Now what?”* |
| Unexpected event VR | Pollution, radiation and toxic substances can increase ROS production. |  | “*No problem, I can handle it*” |
| Dinner  dialogue | In the diet, antioxidants and FA can be introduced in different ways. | To get the best score, I must optimize the intake of both antioxidants and FA. | *“Final test!”* |
| Dinner VR | Some foods are a great source of omega 3 but they aren't palatable.  The use of FA supplements could have contraindications.  Animals fed with omega 3 are a great source of omega 3. |  | *“Ok, I got everything”* |
| Final dialogue |  | Epilogue | *“Well done!”* |


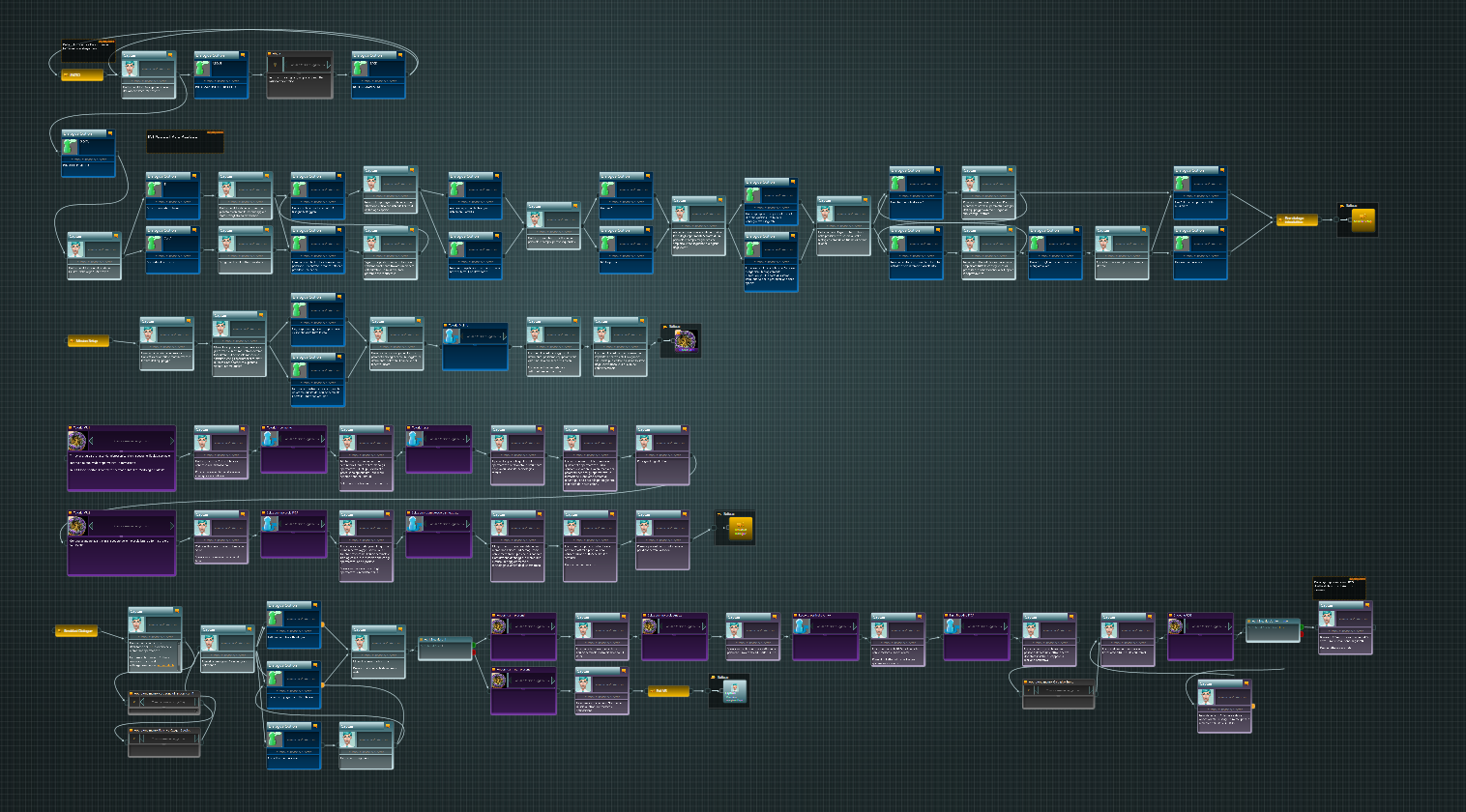


Dialogue structure example (ACT1
